# Emerging Novel Therapeutic Approaches for Treatment of Advanced Cutaneous Melanoma

**DOI:** 10.3390/cancers14020271

**Published:** 2022-01-06

**Authors:** Francesca Comito, Rachele Pagani, Giada Grilli, Francesca Sperandi, Andrea Ardizzoni, Barbara Melotti

**Affiliations:** 1 Medical Oncology Unit, IRCCS Azienda Ospedaliero-Universitaria di Bologna, Via Albertoni, 15-40138 Bologna, Italy; giada.grilli2@unibo.it (G.G.); francesca.sperandi@aosp.bo.it (F.S.); andrea.ardizzoni2@unibo.it (A.A.); barbara.melotti@aosp.bo.it (B.M.); 2 Department of Experimental, Diagnostic and Specialty Medicine (DIMES), University of Bologna, Via Massarenti, 9-40138 Bologna, Italy

**Keywords:** cutaneous melanoma, metastatic melanoma, novel targets, vaccine, immunotherapy, targeted therapy, adoptive cell therapy, anti-LAG-3, bempegaldesleukin, lenvatinib

## Abstract

**Simple Summary:**

The introduction of immune checkpoint inhibitors and targeted therapy for the treatment of unresectable advanced or metastatic melanoma has changed the prognosis of melanoma patients. Five-year overall survival rates for metastatic melanoma have increased from less than 10% up to 40–50%. Despite this revolution in advanced melanoma treatment, many patients do not benefit from currently available therapies or do not achieve durable responses. The introduction of new agents and new strategies of treatment is necessary to improve outcomes.

**Abstract:**

The prognosis of patients with advanced cutaneous melanoma has radically changed in the past decade. Nevertheless, primary or acquired resistance to systemic treatment occurs in many cases, highlighting the need for novel treatment strategies. This review has the purpose of summarizing the current area of interest for the treatment of metastatic or unresectable advanced cutaneous melanoma, including data from recently completed or ongoing clinical trials. The main fields of investigation include the identification of new immune checkpoint inhibitors (anti-LAG3, GITR agonist and anti-TIGIT), adoptive cell therapy, vaccines, engineered TCR therapy, IL-2 agonists, novel targets for targeted therapy (new MEK or RAF inhibitors, HDAC, IDO, ERK, Axl, ATR and PARP inhibitors), or combination strategies (antiangiogenetic agents plus immune checkpoint inhibitors, intra-tumoral immunotherapy in combination with systemic therapy). In many cases, only preliminary efficacy data from early phase trials are available, which require confirmation in larger patient cohorts. A more in-depth knowledge of the biological effects of the molecules and identifying predictive biomarkers remain crucial for selecting patient populations most likely to benefit from novel emerging treatment strategies.

## 1. Introduction

In the recent past, the development of novel treatments has radically modified the prognosis of patients affected with advanced melanoma. Five-year overall survival (OS) rates for metastatic melanoma (MM) have increased substantially from less than 10% up to 40–50% [1]. Immune checkpoint inhibitors (ICI) and targeted therapy represent the two revolutionary treatments. 

Ipilimumab, a monoclonal anti cytotoxic T lymphocyte-associated protein 4 (CTLA-4) antibody, showed a survival benefit over dacarbazine in phase III trials and was the first ICI approved by the Food and Drug Administration (FDA) in 2011 [2]. Subsequently, the anti-programmed death 1 (PD-1) monoclonal antibodies, nivolumab, and pembrolizumab showed superior efficacy compared to ipilimumab and chemotherapy, with lower toxicities. These monoclonal antibodies achieved an overall response rate (ORR) of 40 to 50% [3,4,5] and a five-year OS rate of 41–44% [6,7]. Furthermore, the phase III trial CheckMate 067 showed increased efficacy of the combination of nivolumab and ipilimumab compared with the monotherapies. Higher response rates (nivolumab and ipilimumab vs. nivolumab alone: 58% vs. 44%) and higher long-term overall survival rates were observed, but also a higher rate of toxicity was reported [5,6]. After 6.5 years of follow-up, the median OS was 72.1 months with the combination of nivolumab and ipilimumab, 36.9 months with nivolumab, and 19.9 months with ipilimumab [8]. 

The second breakthrough was the targeted therapy with BRAF and MEK inhibitors for tumors harboring a BRAFV600 mutation. Activating mutations in BRAF are present in about 50% of cutaneous melanoma. Vemurafenib and dabrafenib, two BRAF inhibitors, have significantly improved response rates (approximately 50%), progression-free survival (PFS) and better OS than chemotherapy in patients with metastatic BRAF V600E/K mutant melanoma [9,10]. The association between the BRAF inhibitor and the MEK inhibitor resulted in a notable delay in the onset of resistance, increasing the ORR to 70%, with a longer median PFS than with dabrafenib monotherapy [11]. For MM patients treated upfront with BRAF-MEK-inhibition the median OS is between 22–33 months [12,13,14] and the 5-year OS rate is 34% [13,15]. 

Despite the breakthrough advances in the treatment of MM, improving outcomes for those patients who do not benefit from the current standard of care remains an unmet need. 

## 2. Materials and Methods

This review summarizes clinically relevant data from ongoing clinical trials on the systemic treatment of metastatic or unresectable advanced cutaneous melanoma, including recent data presented at the ASCO (American Society of Clinical Oncology) and ESMO (European Society of Medical Oncology) Annual Meeting. The assessment of treatment efficacy was reported through the median OS, median PFS, duration of response (DOR) and ORR. 

## 3. Results

### 3.1. Novel Immunecheckpoint Inhbitors

#### 3.1.1. Anti-LAG3

**Figure 1:**
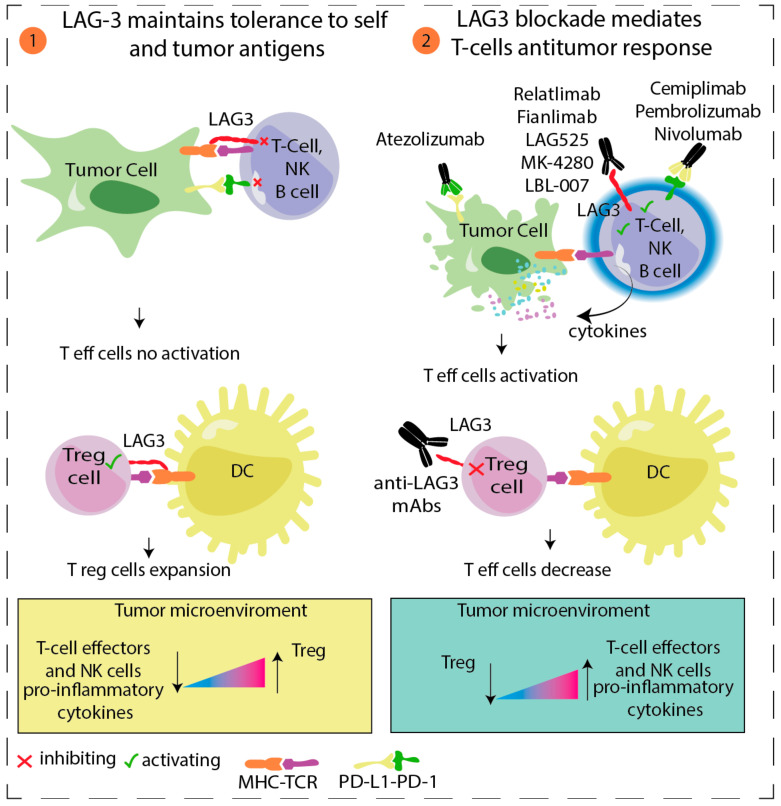
LAG3/MHC Class II Signaling Pathway. (1) LAG-3 is commonly expressed on regulatory T cells (Treg), effector T cells, and NK cells. Its main ligand is MHC class II, which is found upregulated on some tumor cells, tumor-infiltrating macrophages, and dendritic cells (DCs). LAG-3-MHC II interaction promotes inhibition of the responses of effector T cells and promotes the suppressive activity of Treg. (2) Double blocking of LAG3 and PD1 in synergy using antagonist antibodies against LAG3 in combination with PD-1/PD-L1 blockade, represents a novel strategy in oncology. Indeed, co-blocking LAG-3 and PD-1 enhances T-cell effectors and NK cells proliferation, promotes the release of pro-inflammatory cytokine, and inhibits Treg cells [21].

Lymphocyte activation gene-3 (LAG-3) is an inhibitory checkpoint receptor on CD4+ or CD8+ T cells and Tregs, where involvement produces the suppression of T cell activation and proliferation (Figure 1). LAG3 and PD-1 are co-expressed on T cells during T cell receptor signaling and are downregulated after antigen clearance. Persistent stimulation leads to prolonged LAG3 and PD-1 expression and to T cell exhaustion, a possible mechanism of resistance to immunotherapy [16]. Preliminary efficacy data of relatlimab (a human IgG4 LAG-3-blocking antibody) and nivolumab in patients with melanoma that progressed while on or after treatment with anti-PD1 or anti-PDL1 therapy were presented in 2017. Of the 68 treated patients, 57% had previously received anti–CTLA-4 and 46% had received three or more lines of therapy. In 61 efficacy-evaluable patients, the ORR was 11.5% [1 complete response (CR), 6 partial responses (PR)] and the disease control rate (DCR) was 49%. The ORR was ≥3.5-fold higher in patients with LAG-3 expression ≥1% vs. <1%, regardless of PD-L1 expression (NCT01968109) [17]. 

RELATIVITY-047 (CA224-047) is a randomized, double-blind phase II/III study evaluating the combination of relatlimab and nivolumab versus nivolumab alone in patients with previously untreated metastatic or unresectable melanoma. A total of 714 patients were randomized to receive either relatlimab 160 mg + nivolumab 480 mg intravenously every 4 weeks or nivolumab monotherapy 480 mg every 4 weeks. After a median follow-up of 13.2 months, the median PFS in the relatlimab + nivolumab group was significantly longer in the relatlimab + nivolumab arm than in the nivolumab arm (10.1 months vs. 4.6 months, HR, 0.75 [95% CI, 0.6–0.9]; *p* = 0.0055). The 1-year PFS rates were 47.7% and 36% for relatlimab + nivolumab and nivolumab, respectively. The combination was well-tolerated and there were no new safety signals reported for either the relatlimab and nivolumab combination arm or the nivolumab arm [18]. Follow-up for the OS, a secondary endpoint, is ongoing (NCT03470922). Moreover, another phase II trial has been designed to enroll treatment-naive patients with unresectable or metastatic melanoma to receive combined relatlimab and nivolumab after a lead-in arm where patients are randomized to receive relatlimab, nivolumab, or the combination for the first 4-week cycle (NCT03743766) [19]. Currently, relatlimab is also in evaluation in association with ipilimumab in patients with unresectable or metastatic melanoma who have progressed on anti-PD-1 therapy (NCT03978611).

At the 2021 ASCO Annual Meeting, preliminary data on clinical activity from two expansion cohorts of patients with advanced melanoma [anti–PD-(L)1 naive or experienced] who were treated with fianlimab (REGN3767), a human anti-LAG-3 monoclonal antibody, and cemiplimab were presented. A total of 48 patients with advanced melanoma were treated with the combination therapy: 33 were anti–PD-(L)1 naive and 15 were anti–PD-(L)1 experienced. By investigator assessment, ORR was 63.6% (3 CR and 18 PR) for anti–PD-(L)1 naive patients and 13.3% (1 CR and 1 PR) for anti–PD-(L)1 experienced patients. The median PFS and median DOR for the anti–PD-(L)1 treatment naive cohort have not been reached. The safety profile of fianlimab + cemiplimab is similar to that documented with cemiplimab monotherapy and other anti–PD-1s, with the exception of a higher rate of adrenal insufficiency (8.3%) [20]. 

Other LAG-3-modulating agents have become subjects of clinical trials. For example, LAG525, MK-4280, and LBL-007 are currently undergoing phase I/II trials as a monotherapy or in combination with anti-PD-1 (NCT02460224; NCT02720068; and NCT04640545 phase I study).

#### 3.1.2. GITR Agonist

**Figure 2:**
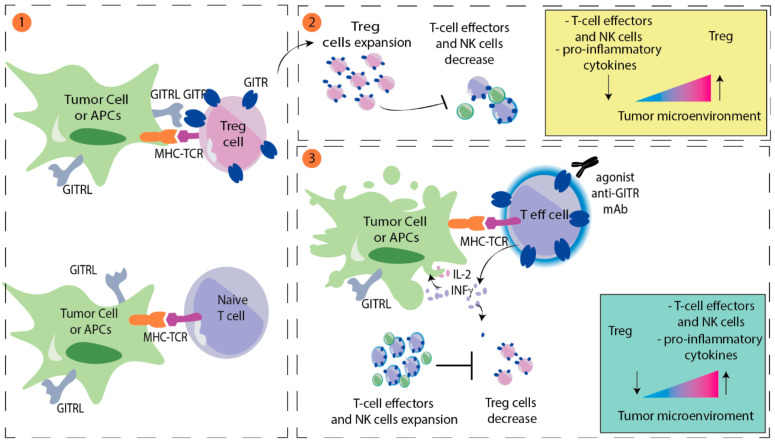
Model for GITR modulation of antitumor immunity. (1) GITR ligand (GITRL) is mainly expressed on tumor cells or antigen presenting cells (APCs). High levels of GITR are normally expressed on regulatory T cells (Treg). In contrast, on effector T cells, GITR expression is upregulated following activation by TCR-MCH interaction. (2) GITR engagement on Treg cells promotes Treg cells activation and proliferation and inhibits proliferation of effector T and NK cells. (3) Conversely, GITR-GITRL interaction on effector T cells promotes antitumor activity of T cells by enhancing CD4+ and CD8+ T-cell proliferation and downregulates the immunosuppressive activity of Tregs. Activation of effector T cells can also be stimulated using agonists anti-GITR, such as BMS-986156 or TRX518.

The glucocorticoid-induced TNF receptor (GITR) is a member of the tumor necrosis factor (TNF) receptor superfamily. GITRs are constitutively expressed at high levels on regulatory T cells (Treg) and at low levels on resting CD4+ T cells, CD8+ T cells, and natural killer (NK) cells [22,23]. After T-cell activation, GITR expression is upregulated on CD4+ and CD8+ T cells and NK cells [24]. The GITR ligand is expressed at low levels by antigen-presenting cells (APC) and is upregulated upon activation [25]. Activating the GITR pathway promotes antitumor activity of T cells by enhancing CD4+ and CD8+ T-cell proliferation and effector functions and may protect T cells from activation-induced cell death [26]. GITR ligation enhances T-cell survival by upregulating IL2, IL2 receptor alfa, and IFN gamma [27]; activation downregulates the immunosuppressive activity of Tregs [28] (Figure 2). 

There are several anti-GITR agents being investigated in early phase clinical trials. BMS-986156 is a GITR agonistic monoclonal antibody which was tested alone and in combination with nivolumab in advanced solid tumors (including seven melanoma patients) in a phase I/IIa study. Overall, the combination was well tolerated with no dose-limiting toxicities. No responses were seen with BMS-986156 single agent, while an ORR of 9.0% (18 of 200) was observed in the patient cohort evaluable for response that received the combination of BMS-986156 plus nivolumab [29]. MK-4166 is a humanized IgG1 agonist targeting GITR. In the phase I study (NCT02132754) of MK-4166 as a monotherapy or in combination with pembrolizumab, 20 patients affected with advanced melanoma were enrolled in the combination arm. For ICI-naive melanoma patients (*n* = 13), the ORR was 61.5%, including five CRs and three PRs. While for seven patients previously treated with ICI, no response was observed [30]. 

#### 3.1.3. Anti-TIGIT

**Figure 3:**
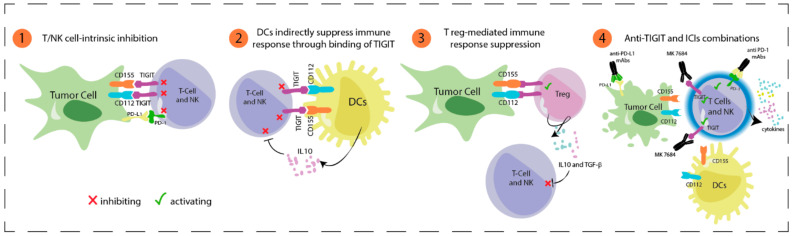
TIGIT in cancer immunotherapy (1) TIGIT is expressed on effector T and NK cells. TIGIT binds two ligands, CD155 and CD112, that are expressed on tumor cells and dendritic cells (DCs). (2) TIGIT-CD155 interaction in DCs promotes the release of anti-inflammatory cytokines such as IL-10, which impairs T effector and NK cells activation. (3) Interaction between TIGT-CD155/CD112 in regulatory T cells (Treg) facilitates suppression of antitumor immune response by releasing cytokines into the tumor microenvironment, such as IL-10 and TGF-β, which have an inhibiting effect on T effector and NK cells. (4) Blocking TIGIT with monoclonal antibodies (mAbs), alone or in combination with anti-PD1, can restore T effectors and NK cells activity in cancer patients.

T cell immunoreceptor with immunoglobulin and ITIM domain (TIGIT) is another promising new target for cancer immunotherapy. TIGIT binds to two ligands, CD155 and CD112, that are expressed by tumor cells and APCs to down-regulate T cell and NK cell functions [31] (Figure 3). Double PD-1 and TIGIT inhibition enhances tumor antigen-specific CD8+ T cell expansion and activity in vitro, and increases proliferation and function of tumor antigen-specific CD8+ T cells and tumor infiltrating lymphocytes (TILs) isolated from patients with melanoma, as compared with single inhibition [32]. Two phase I/II studies are currently recruiting patients with advanced melanoma. In the sub-study 02A of the KEYMAKER-U02, 200 patients with PD-1 refractory melanoma are randomized to receive vibostolimab (MK-7684), a TIGIT blocking humanized IgG1 monoclonal antibody, or lenvatinib with pembrolizumab and quavonlimab (anti-CTLA-4) (NCT04305041). In the sub-study 02B KEYMAKER-U02, previously untreated advanced melanoma patients will receive pembrolizumab plus vibostolimab or pembrolizumab alone (NCT04305054). 

Ongoing clinical trials with novel immune checkpoint inhibitors are listed in Table 1.

### 3.2. Adoptive Cell Therapy

Adoptive cell therapy consists of the isolation of TILs from the excised tumor, expansion of these cells by interleukin-2 (IL-2) treatment and re-infusion into lympho-depleted patients with treatment of IL-2 in addition. The first employment of adoptive cell therapy with TILs was performed by Rosenberg and colleagues. Patients received a single dose of cyclophosphamide, infusion of autologous TILs and a high dose (HD) of IL-2. The ORR was 55% (11/20), including one CR [33]. Following studies modified chemotherapy regimen to obtain lymphodepletion without myeloablation, using cyclophosphamide and fludarabine [34]. In 2019, Dafni and colleagues published a meta-analysis with the aim of evaluating efficacy in patients who previously received treatments for advanced cutaneous melanoma. The pooled overall ORR estimate was 41% and the overall CR rate was 12%. Most of HD-IL-2 complete responders (27/28) maintained remission after a median follow-up of 40 months [35,36]. These results indicate that TIL therapy affords the potential for durable clinical advantage, even in patients with few therapeutic options. Borch et al. presented the results of their study using T-cell therapy in ICI resistant melanoma. Of 53 evaluable patients, 6 patients achieved CR and 14 achieved PR, hence the overall ORR was 38%. After a median follow-up time of 60 months, for the overall cohort the median OS (mOS) was 15.9 months, and the 3-year survival rate was 29%. The median PFS (mPFS) was 3.7 months, but only a few patients progressed later than 12 months after treatment. Of the 20 responder patients (CR or PR), 13 (65%) were still alive. Hence, the mOS was not reached and the mPFS was 19.1 months for this subgroup. The 3-year survival rate for complete responders was 100% [37]. 

C-144-01 is a recent phase II open-label study of the efficacy and safety of lifileucel (LN-144) in patients with unresectable MM who have progressed on ICI and BRAF/MEK inhibitors, in BRAF V600 mutant. Therapy consisted of one week of nonmyeloablative lymphodepletion (using 2 days of cyclophosphamide and 5 days of fludarabine), a single lifileucel infusion, and up to six IL-2 doses. Sixty-six patients heavily pretreated (3.3 mean prior therapy lines) and with high baseline tumor burden were enrolled. The ORR was 36.4% (3 CR, one PR converted to a new CR at 24 months, 21 PR) [38]. After a median follow-up of 28 months, the median DOR was not reached, which is positively associated with primary resistance to prior anti-PD1 therapy and with a shorter duration of anti-PD1 therapy (NCT02360579) [39]. A randomized controlled phase III study is currently assessing if TIL infusion preceded by non-myeloablative chemotherapy and followed by HD IL-2 can produce an improved PFS as compared with ipilimumab in 168 MM patients (NCT02278887). DELTA-1 is an ongoing phase II clinical trial to assess the efficacy and safety of ITIL-168, a cell therapy derived from patient’s own TIL cells, in subjects with advanced melanoma who have previously been treated with a PD-1 inhibitor (NCT05050006).

Adoptive cell therapy has been combined with ipilimumab in a phase I trial that enrolled 13 patients. Five (38.5%) patients had an objective response. After a 1-year follow-up, four (4/5) patients continued in objective response and one patient obtained CR at 52 months [40]. In a pilot trial, the association of vemurafenib with adoptive cell therapy in BRAF mutant MM showed clinical efficacy. The ORR was 64% (7/11), 18% (2/11) had a CR for 3 years (one response ongoing at 46 months) [41]. 

Furthermore, numerous early phase trials are ongoing to evaluate the safety and feasibility of a combination therapy of a TILs transfer with nivolumab in patients with pretreated metastatic melanoma (NCT03475134, NCT04165967, NCT03374839, NCT03638375). 

Ongoing clinical studies with adoptive cell therapy are listed in Table 2.

### 3.3. Vaccines

The promise of cancer vaccines is that vaccines induce targeted, tumor-specific immune responses with long-term memory in cases of recurrence or metastasis, with a low risk of toxicity overall. Vaccines may use as an antigen whole tumor cells, RNA or DNA, single or multiple peptides, or APCs displaying the target antigen [42]. 

T cells bind cancer neoantigens, a group of Human Leukocyte Antigen (HLA)-bound peptides that result from tumor-specific mutations, and play their role in anti-tumor immunity [43]. 

#### 3.3.1. Peptide Vaccines

Peptide vaccines can be synthesized as short or long with single- or multi-peptide mixtures. Peptides are weakly immunogenic when naked peptide is used; however, their use in combination with vaccine adjuvants or immune therapies induces potent, and frequently durable, T cell responses [44]. 

Telomerase is expressed in tumor cells at every phase of cancer evolution and implicated in human cell immortalization and cancer cell pathogenesis. UV1, a telomerase peptide-based vaccine, includes three long peptides forming a 54 amino acid sequence in the catalytic unit of the reverse transcriptase subunit of telomerase (hTERT). UV1 includes both CD4 and CD8 epitopes. It has been shown to be immunogenic in 78% of HLA unselected patients across three completed phase I studies. It induces proliferation of hTERT specific CD4+ T cells [45]. In a phase I/IIa, single institution study (NCT02275416), 12 patients with MM received ipilimumab plus repeated UV1 vaccinations, with Granulocyte-Macrophage Colony Stimulating Factor (GM-CSF) as an adjuvant. Nine patients were evaluable for tumor response. As best overall response, one patient obtained CR, three obtained PR, two obtained stable disease (SD), and three obtained progression disease (PD). Patients, who were not evaluable according to RECIST 1.1, progressed clinically. One patient had an ongoing CR at the 5-year follow-up analysis. At the median follow-up of 61 months, mOS was not reached. The 2-year OS rate was 75%, the 3-year OS rate was 67%, and the 5-year OS rate was 50%. The median PFS was 6.7 months. The 1-year PFS was 33% and the 2-years PFS was 25% [46]. The combination of UV1 plus nivolumab and ipilimumab is under evaluation (NCT04382664). In another phase I clinical trial, telomerase vaccine UV1 was evaluated in combination with pembrolizumab in patients with advanced ICI-naive melanoma. Thirty patients were enrolled and results from cohort one (20 patients) were presented at ASCO 2021. Among the 20 patients treated with UV1 300 mcg + GM-CSF 37.5 mcg, the RR was 60%: six (30%) achieved a CR, six (30%) a PR, and one (5%) an SD. After a median follow-up of 21.2 months, the mPFS was 18.9 months and 80% of the patients were alive. Results from cohort two (10 patients, treated with UV1 300 mcg + GM-CSF 75 mcg) will be presented after reaching the one-year follow-up (NCT03538314) [47]. 

The IDO (Indoleamine 2,3-dehydrogenase)/PD-L1 (IO102/IO103) peptide vaccine is a novel immune-modulatory vaccine consisting of IDO- and PD-L1 peptide sequences designed to target the immunosuppressive mechanisms mediated by key immunosuppressive proteins such as IDO and PD-L1. A phase I/II study evaluated the IDO/PD-L1 peptide vaccine plus nivolumab in patients with progressing MM. An ORR of 79% was reached (CR 45%, PR 34%); the ORR was 94% and 62% in PD-L1 positive and negative patients, respectively. The mPFS was 25.6 months (NCT03047928) [48]. 

Numerous phase I trials with a personalized neoantigen cancer vaccine in combination with anti-PD-1 are ongoing (NCT04364230, NCT04072900, NCT03929029). 

#### 3.3.2. Dendritic Cell Vaccines

Vaccination with ex-vivo tumor antigen-loaded autologous dendritic cells (DCs) offer another treatment option [49]. A phase I clinical trial evaluated the use of myeloid DCs activated and loaded with HLA-A*02:01-restricted melanoma peptides gp100 and tyrosinase ex vivo. Of 14 untreated stage IV melanoma patients, two patients obtained the objective response. The mPFS was 17.6 months for three patients who developed immunological response, while the mPFS was 2.3 months for patients without functional T cells in their blood (*p* = 0.019, HR 0.15; 95% CI, 0.04–0.57). The mOS of all patients was 13.3 months [50]. Vaccination with the DCs vaccine in combination with ICI monotherapy may provide clinical benefits and is currently in evaluation (NCT02678741, NCT03092453) [51]. 

#### 3.3.3. RNA Vaccines

The Lipo-MERIT trial is a multicenter, open-label, dose-escalation, phase I trial which represents the first experience in vivo of RNA vaccines application (NCT02410733). FixVac (BNT111) consists of RNA-LPX, a single-stranded antigen-encoding RNA in liposomal formulation. RNA encodes four tumor-associated antigens (TAAs)—New York oesophageal squamous cell carcinoma 1 (NY-ESO-1), melanoma-associated antigen A3 (MAGE-A3), tyrosinase, and trans-membrane phosphatase with tensin homology (TPTE)—that present limited normal tissue expression, elevated immunogenicity, and prevalence in melanoma. The LPX protects RNA from extracellular ribonucleases and selectively delivers the RNA into APCs in lymphoid compartments. The engulfed RNA is translocated to the cytoplasm, where it is translated by the ribosome complex into the encoded protein. The close proximity of APCs to T cells in lymphoid tissues is the ideal microenvironment for efficient priming and amplification of CD8+ and CD4+ T cell responses [52]. Concurrently, RNA-LPX activates APCs via signaling through toll-like receptors (TLRs), resulting in a pulsatile release of pro-inflammatory cytokines. Sahin et al. presented the results of an exploratory interim analysis of 89 patients with advanced melanoma that express at least one of the four targeted TAAs. The best objective response was assessed in 42 patients with measurable disease. Forty-one were previously treated for MM; 35 of these had received both anti-PD1 and anti-CTLA4 antibodies. In the FixVac monotherapy group (*n* = 25), three patients achieved a partial response and seven had stable disease. One patient showed a complete metabolic response of metastatic lesions in 18fluorodeoxyglucose—Positron Emission Tomography/Computerized Tomography imaging. In the FixVac with anti-PD1 combination arm, six out of 17 patients experienced a partial response (35%), and two patients had stable disease [53]. A randomized phase II trial has been evaluating BNT111 and cemiplimab in combination or as single agents in 120 patients affected with anti-PD1-refractory or relapsed metastatic or advanced melanoma (NCT04526899). 

Ongoing clinical studies with vaccines are listed in Table 3.

### 3.4. Engineered TCR Therapy

The structure of a chimeric antigen receptor (CAR) consists of a single-chain variable fragment derived from a monoclonal antibody targeting a cancer-specific antigen, intracellular segment, signaling domain derived from T-cell receptor (TCR), and one or more co-stimulatory sequences (CD28, OX40 or 4-1BB) [54]. CAR are able to bind both antigens on cancer cells and T cells activating functions [55]. CAR T-cell therapy had success in treating patients with hematologic diseases, however, for solid tumors such as melanoma, it obtained low response rates (19% for CARs binding gp100 and 30% for CARs binding DMF5) [56,57]. Several studies are ongoing to evaluate the use of CAR T-cell therapy in patients with advanced melanoma (NCT03893019; NCT04119024; NCT03649529; NCT02107963).

Ongoing clinical studies with engineered TCR therapy are listed in Table 4.

### 3.5. Interleukin-2

IL-2 is an endogenous agonist of the IL-2 signaling pathway. The immunostimulatory and immunosuppressive effects of the IL-2 pathway are mediated through IL-2 binding to its receptors (IL-2Rs), which are composed of different combinations of subunits including α (CD 25), β (CD122), and γ (CD132) [58]. HD IL-2, such as aldesleukin, which binds to both the intermediate-affinity IL-2Rβγ receptor and the high-affinity IL-2Rαβγ receptor, has been approved for the treatment of MM in many countries worldwide [58]. Treatment with HD IL-2 in patients with metastatic melanoma has demonstrated an ORR up to 15%, with approximately 5% of patients developing a CR and a part of the responders showing durable long-term responses [59]. However, treatment with HD IL-2 is associated with a high risk of serious adverse events, including capillary leak syndrome, hypotension, respiratory failure, oliguria, and anuria [60]. 

An ongoing phase II study has the aim of exploring the efficacy of HD IL-2 in combination with low dose ipilimumab followed sequentially by nivolumab in patients with unresectable or metastatic melanoma who had progressed on prior anti-PD1 therapy (NCT04562129).

Bempegaldesleukin (NKTR-214 or BEMPEG), a novel CD122-preferential IL-2 pathway agonist, produces sustained signaling through the IL-2βγ receptor, which activates effector T and NK cells over regulatory T cells [59] and increases proliferation of TILs and PD-1 expression on effector T cells in the tumor microenvironment. The safety and clinical activity of the combination of bempegaldesleukin plus nivolumab have been evaluated in PIVOT-02 (NCT02983045), a global, multicenter phase I/II study of multiple solid tumors. A total of 41 previously untreated patients with unresectable or metastatic melanoma received BEMPEG 0.006 mg/kg plus nivolumab 360 mg once every 3 weeks for 2 years; 38 patients were efficacy evaluable. The ORR was 52.6% and 13 patients achieved CRs (34.2%). Responses were durable and deepened over time. After a median follow-up of 29 months, the median DOR and mOS were not reached. The median PFS was 30.9 months. The PFS and OS rates at 36 months were 45.5% and 70.9%, respectively [61]. The combination was well-tolerated and had a good safety profile. The more common adverse events included flu-like symptoms, rash and fatigue. 

The phase III PIVOT IO 001 study is ongoing with the aim to confirm the PIVOT-02 phase II study findings (NCT03635983) [59]. PROPEL, a phase I/II trial is ongoing to evaluate the efficacy of NKTR-214 in combination with pembrolizumab in patients with advanced solid tumors (NCT03138889) [62].

NKTR-262 is a novel toll-like receptor (TLR) 7/8 agonist constructed to stimulate tumoral antigen-specific immunity. The combination of NKTR-262 and NKTR-214 stimulates innate and adaptive immunity, providing an abscopal response and systemic antitumoral immunity. The phase Ib/II REVEAL trial is ongoing to evaluate intratumoral administration of NKTR-262 and intravenous administration of NKTR-214. Initial dose levels of NKTR-262 with fixed-dose NKTR-214 were well-tolerated with early evidence of clinical activity. These results support the following evaluation of the combination with or without nivolumab (NCT03435640) [63]. 

Nemvaleukin (ALKS 4230) is an investigational engineered fusion protein consisting of modified IL-2 and the high affinity IL-2 alpha receptor chain. The selectivity of nemvaleukin is designed to increase the anti-tumoral effects of IL-2 therapy and to reduce certain limits of IL-2 use. Besides, it preferentially stimulates CD8+ T cells and NK cells with minimal expansion of regulatory T cells [64]. ARTISTRY-6, a phase II study, is evaluating the efficacy of ALKS 4230 monotherapy administered subcutaneously in patients with advanced cutaneous or mucosal melanoma who have previously received anti-PD-(L)1 therapy (NCT04830124).

Hu14.18-IL2 is the combination of humanized anti-GD2 (disialoganglioside) antibody conjugated to IL-2. A phase I/II trial (NCT03958383) has been designed to determine the safety and efficacy of intratumoral hu14.18. This clinical trial includes four cohorts: hu14.18-IL2 alone, with radiation therapy, after radiation therapy and in combination with nivolumab, and after radiation therapy and in combination with nivolumab and ipilimumab in patients with pretreated advanced or unresectable melanoma.

Ongoing clinical studies are listed in Table 4.

### 3.6. Targeted Therapy

#### 3.6.1. New MEK or RAF Inhibitors

There is an unmet medical need for new targeted therapy opportunities in patients whose tumors harbor an NRAS mutation, occurring in 15–25% of patients with melanoma and producing the activation of Ras/Raf/MEK/ERK signaling pathway. The presence of NRAS mutation has been recognized as an independent poor prognostic factor in MM [65]. Up to this point, no drugs have been approved to specifically treat melanoma patients with NRAS mutation or amplification. Binimetinib, a MEK inhibitor, showed only a modest PFS benefit [2.8 vs. 1.5, HR 0.62, 95% confidence interval (CI) 0.47–0.80, *p* < 0.001] in NRAS-mutated melanoma who were treatment naive or had progressed on or after prior immunotherapy, compared with dacarbazine in a randomized phase III clinical trial (NEMO). There was no improvement in overall survival (HR 1.00, *p* = 0.499) [49,66]. 

Pimasertib, another MEK1/MEK2 inhibitor, has been compared with dacarbazine in a phase II trial in patients with untreated NRAS-mutated melanoma. The mPFS was 13 weeks and 7 weeks for pimasertib and dacarbazine, respectively (HR 0.59; *p* = 0.0022). The ORR was improved with pimasertib (27 vs. 14%). However, the OS was similar between two groups (9 versus 11 months, respectively; HR 0.89), but the crossover was permitted and 64% of patients treated with dacarbazine received pimasertib [67]. 

FCN-159 is another new MEK inhibitor. Its selectivity is more than 10 times higher than that of trametinib against activated MEK1 and MEK2. A phase Ia/Ib, open label, dose-escalation study with expansion cohort is ongoing to evaluate the safety and efficacy of FCN-159 in 37 patients with NRAS aberrant unresectable or metastatic melanoma (NCT03932253). 

Moreover, belvarefenib, a selective RAF dimer inhibitor, in combination with cobimetinib demonstrated some efficacy in NRAS mutant melanoma treated with prior ICI therapy in a phase Ib study (NCT03284502) [68] and it is currently in evaluation as a single agent or in combination with cobimetinib or with cobimetinib plus atezolizumab in 83 patients with NRAS-mutant advanced melanoma who were previously treated with anti-PD-1/PD-L1 therapy (NCT04835805).

LXH254 is a potent inhibitor of BRAF and CRAF, which showed activity in models harboring BRAF alterations, including atypical BRAF alterations co-expressed with mutant K/NRAS, and NRAS mutants [69]. The combinations of LXH254 and trametinib or the ERK inhibitor, LTT462, are currently in evaluation in patients with previously treated BRAF V600 or NRAS mutant melanoma (NCT04417621). 

#### 3.6.2. CDK4/6 Inhibitors

Cyclin-dependent kinase (CDKs) are a family of serine/threonine kinases with a regulatory role at checkpoints during the cell cycle, in combination with cyclin proteins. Hyper-activation of the CDK4/6-Rb-p16INK4A pathway is common and implicated in approximately 90% of melanomas [70]. 

An interesting potential role for CDK inhibitors in melanoma is to use these molecules in association with BRAF and MEK inhibitors. Preclinical models have showed that the addition of ribociclib to encorafenib delays the onset of BRAF resistance. Taylor et al. presented the results from a small clinical trial. Nine patients were response evaluable at the time of publication. Two patients achieved PR and six patients achieved SD with high incidence of toxicities [71]. The use of both MEK and CDK4/6 inhibitors may suppress activated MAP kinase pathway and cell cycle checkpoint dysfunction in NRAS mutant melanoma, increasing antitumoral efficacy [72], by upregulating activity of the RTK-RAS-RAF and RTK-PI3K-AKT signaling cascade. Binimetinib plus ribociclib showed a favorable efficacy and safety profile in a phase Ib trial of 16 patients with metastatic NRAS-mutant melanoma. Four patients (25%) achieved a partial response and seven patients (44%) achieved stable disease, resulting in a disease control rate of about 70% [73]. A phase Ib/II study (NCT01543698) evaluated triple combination therapy with encorafenib, binimetinib and ribociclib in patients with BRAF V600 mutant melanoma naive to prior BRAF inhibitor treatment and with high tumor burden. ORR was 52.4%, including 4 CR, 18 PR and 15 SD. However, the median PFS was inferior than that previously obtained with the combination of encorafenib and binimetinib (9.2 vs. 11.3 months) and this combination seems to increase toxicity [74]. 

Based on the positive results of previous use of palbociclib in monotherapy in pretreated patients, harboring CDKN2A loss [75], CELEBRATE study was designed. This is a phase Ib, dose escalation study with expansion phase to assess the safety, tolerability, and pharmacokinetics of the encorafenib, binimetinib and palbociclib combination in BRAF-mutant MM (NCT04720768). 

Preclinical models have shown that CDK4/6 inhibitor produce modifications in the tumoral microenvironment and tumor-secreted cytokines resulting in increased T-cell activity and in reducing regulatory T cells activity. This immunomodulating effect could increase the efficacy of immunotherapy. Some promising results of efficacy have been shown in vitro and a phase I/II clinical trial of immunotherapy and CDK4/6 inhibitor combinations is underway (NCT02791334) [70]. 

#### 3.6.3. ERK Inhibitors

The MAP/ERK signaling pathway has a key role in tumor development by promoting cell proliferation and migration. Inhibitors of downstream components in MAP kinase pathway could provide more complete blockade of the pathway. Particular attention was paid to ERK (Extracellular signal-related kinases) inhibitors. In preclinical models ERK inhibitors have wider efficacy than MEK inhibitors. Ulixertinib (BVD-523) has shown early evidence of clinical activity in a phase I trial, that included both patients with NRAS-mutant melanoma and BRAF-mutant melanoma which progressed on or were refractory to BRAF and/or MEK inhibitors [76]. An expanded access program is open to provide ulixertinib for compassionate use for treatment of advanced solid tumors with MAPK pathway-alteration, including but not limited to KRAS, NRAS, HRAS, BRAF, MEK, and ERK mutations, after the exhaustion or after incomplete response to available therapies (NCT04566393). Other ERK inhibitors are being investigated in early phase trials: LY3214996 (NCT02857270), ASN007 (NCT03415126) [77].

#### 3.6.4. HDAC Inhibitors

Treatment with BRAF and MEK inhibitors in melanoma is characterized by the development of resistance related to the onset of secondary mutations. Preclinical and clinical studies have suggested that short-term treatment with the HDAC (Histone Deacetylase) inhibitor vorinostat can eliminate cells harboring these secondary mutations causing resistance. Huijberts et al. have conceived phase I/II study to determine the efficacy of sequential treatment with vorinostat and BRAF inhibitor/MEK inhibitor in resistant BRAF V600E mutant melanoma (NCT02836548) [78]. 

Epigenetic modifiers could induce changes in tumor microenvironment through the increase of effector T cells function and of antigen expression. HDAC inhibitors prevent activation-induced cell death of T cells, and preclinically HDAC inhibitors in combination with ICI may inhibit T cell death and increase anti-tumor effect [79]. Several trials are evaluating the effects of the adjunct of HDAC inhibition to PD-1 blockade. Domatinostat, a class I-selective oral HDAC inhibitor, enhances the expression of genes known to reinforce immune responses against tumors [80]. Preliminary data of the open label phase Ib/II study SENSITIZE, that combines domatinostat with pembrolizumab in patients with primary refractory or non-responding to anti-PD-1 therapy are encouraging (NCT03278665) [81]. Likewise, entinostat, another class I-selective HDAC inhibitor, in combination with pembrolizumab demonstrated promising activity, through change of the immunosuppressive tumor microenvironment to restore inflammation. ENCORE-601 enrolled patients with unresectable or MM, previously treated with an anti PD-(L)1, and experienced progression on or after therapy. 6 of 34 (18%) patients achieved a confirmed PR and 3 have been on treatment more than a year [82]. On the contrary, a phase I trial with panobinostat, another HDAC inhibitor, did not show to increase response in combination with ipilimumab [83]. 

HBI-8000 is a class I selective oral HDAC inhibitor, which was tested in combination with nivolumab in 49 melanoma patients. 31 not previously treated with anti-PD1 patients were evaluable for response, of which 23 patients achieved objective responses (4 CR, 19 PR; ORR 74%), 5 SD (disease control rate 90%), and 3 PD. Median time to response was 1.9 months. At a median follow-up of 8.9 months, the median duration of response and median progression-free survival had not been reached (NCT02718066) [84]. On the basis of this result, a phase III study of HBI-8000 or placebo combined with nivolumab in patients with unresectable or MM not previously treated with PD-1 or PD-L1 inhibitors is ongoing (NCT04674683). 

#### 3.6.5. IDO Inhibitors

Indoleamine-2,3-dioxygenase (IDO) is an intracellular heme-containing enzyme that initiates the first and rate-limiting step of tryptophan degradation along the kynurenine pathway. Through the process of tryptophan depletion, IDO exploits an immunosuppressive effect, facilitating immune escape of tumors [85]. The expression of IDO was associated with negative outcome in melanoma. Epacadostat is a potent and specific IDO1 inhibitor that is being evaluated in combination with ICI. A phase I/II study assessing epacadostat plus ipilimumab in patients with unresectable or MM showed an ORR of 75% (9/12 patients) with one complete response [86]. Epacadostat in combination with pembrolizumab showed promising antitumour activity in the phase I/II ECHO-202/KEYNOTE-037 study [87]. However, in the phase III trial (ECHO-301/KEYNOTE-252), comparing epacadostat and pembrolizumab with pembrolizumab alone, no significant differences were found between the treatment arms for PFS (median 4.7 months, for epacadostat plus pembrolizumab vs. 4.9 months, for placebo plus pembrolizumab; HR 1.00, *p* = 0.52) or OS (median not reached in either group; HR 1.13, *p* = 0.81), after a median follow-up of 12.4 months [88]. The encouraging mPFS of patients in the phase I/II ECHO-202 report was probably not due to the addition of epacadostat, but instead to the inclusion of a particularly favorable population. 

Similarly, epacadostat was combined with nivolumab in a phase I/II study (ECHO-204) of patients with advanced solid tumors. Fifty patients with advanced melanoma were enrolled, of which 40 were treatment naive for advanced disease. In treatment-naive patients, the ORR was 65% (26/40; 8 CR, 18 PR) and the DCR was 80% (32/40); the rate of PFS at 12 months was 63% (median not reached) and the OS rate at 12 months was 92% (median not reached) [89].

Another IDO-1 inhibitor, BMS-986205, has also been evaluated in combination with nivolumab. A phase I/II trial showed antitumor activity with a favorable safety profile [90]. However, the phase III study stopped enrollment prematurely due to failure of this combination in another phase III trial in solid tumors (NCT03329846). The discordant results underline the need to better understand the biological effects of the molecules and recognition of relevant biomarkers capable of selecting patient subgroups most likely to benefit from treatment.

Indoximod, another IDO inhibitor, has been tested in combination with ipilimumab, nivolumab or pembrolizumab in a phase II study. Eighty-nine patients with non-ocular melanoma received indoximod in combination with pembrolizumab. The ORR was 51% (CR 20%) and the disease control rate was 70%. The median PFS was 12.4 months (95% CI 6.4 to 24.9). The dual treatment was well tolerated, and side effects were similar to what was expected from single agent pembrolizumab [91]. 

#### 3.6.6. Anti-Angiogenic Therapy

The vascular endothelial growth factor (VEGF) plays an important immunosuppressive role in the tumor microenvironment [92]. Preclinical data suggest that melanoma angiogenesis promotes resistance to MAPK-pathway inhibitors (MAPKi) and immune checkpoint inhibitors. 

Lenvatinib is a potent inhibitor of VEGFR 1-3, FGFR 1-4, PDGFRα, RET, and KIT. KEYNOTE-146 is a phase Ib/II trial that combined lenvatinib with pembrolizumab in patients with advanced melanoma previously treated with 0–2 therapies. Overall, the ORR was 48%, median DOR was 12.5 months, mPFS was 5.5 months, and 1y-PFS rate was 34.7% [93]. A second phase II trial (LEAP-004) assessed the efficacy of this combination in patients that progressed on anti-PD-(L)1 with or without concomitant anti-CTLA4 therapy in 103 patients. After a median follow-up of 15.3 months, the ORR was 21.4% (three CRs) in the overall population, 33.3% in 30 patients progressing on anti-PD-(L)1 + anti-CTLA-4, 18.2% in patients who had received anti–PD-1/L1 only in the adjuvant setting (*n* = 11), 22.6% in patients with primary resistance (*n* = 62), and 22.7% in patients with secondary resistance (*n* = 22). The median PFS and OS in the overall population were 4.2 months and 14 months, respectively; 12-month PFS and OS estimates were 17.8% and 54.5% [94,95]. These results support lenvatinib plus pembrolizumab as a potential therapeutic option for this population with high unmet needs. The same combination is currently being tested as a first-line treatment in the LEAP-003 trial (NCT03820986). 

Axitinib targets VEGFR1-3, cKIT, and PDGFR. Even axitinib has been studied in combination with immune agents. The rationale is that this molecule is able to decrease hypoxia in the TME and is, therefore, able to re-sensitize melanoma tumors to anti-PD1 therapy in patients who have progressed on previous anti-PD1 therapy alone or in combination with anti-CTLA4 therapy [92]. A phase II trial combining axitinib with nivolumab is ongoing (NCT04493203). 

Bevacizumab was the first anti-angiogenic therapy used in patients with advanced melanoma. This drug has been used in monotherapy or in combination with other anti-angiogenic treatments (sorafenib or dacarbazine) demonstrating, although only a little, promising clinical efficacy [96,97]. In 2014, Hodi et al. used bevacizumab in combination with ipilimumab. The combination reveals that VEGF-A blockade exerts influence on inflammation, lymphocyte trafficking, and immune regulation. The best overall response included eight PR, 22 SD (46 patients enrolled), and a disease-control rate of 67.4%. The median OS was 25.1 months [98]. Two phase II studies are ongoing to evaluate the efficacy in combination with ICI (ipilimumab NCT01950390, atezolizumab NCT04356729). 

#### 3.6.7. AXL Inhibitors

Upregulation of the receptor tyrosine kinase Axl has been associated with reduced efficacy of immune checkpoint inhibitors and the onset of resistance to BRAF targeted therapy. Bemcentinib (BGB324) is a first selective inhibitor of Axl. BGBIL006 is an ongoing, open label phase Ib/II trial designed to assess if a combination of bemcentinib and pembrolizumab or dabrafenib plus trametinib improves the ORR and DOR compared with pembrolizumab or dabrafenib plus trametinib alone (NCT02872259) [99]. Another approach that is currently being pursued is the development of antibody–drug conjugates to deliver a chemotherapy payload with an AXL-directed antibody (NCT02988817). 

#### 3.6.8. PARP Inhibitors

Analysis of The Cancer Genome Atlas (TCGA) and the Cleveland Clinic’s Gross Family Melanoma Registry reveals that a significant proportion (~40%) of melanoma patients possess somatic (31.6%) or germline (TCGA: 4.2%; registry: 8.3%) mutations in homologous recombination (HR) repair genes, which may serve as a therapeutic target. The most commonly aberrant gene was ARID2, followed by ARID1A, FANCA, ATM, BRCA1, ATRX and BRCA2, ATR, BRCA1, and BRIP1 [65]. 

A phase II trial is recruiting MM patients with HR mutation or alteration who have had disease progression on previous immunotherapy, BRAF inhibitor, or both, to evaluate the efficacy and safety of monotherapy with niraparib (NCT03925350). Furthermore, the use of PARP inhibitors in combination with ICI may have a synergistic immunomodulatory and antitumor effect. A phase II, single arm, open label trial is ongoing to evaluate the efficacy of combination of nivolumab plus talazoparib in unresectable or MM patients harboring a somatic or germline mutation or deletion in BRCA or BRCAness (genes including ARID1A/B/2, ATM, ATR, BAP1, BARD1, BLM, BRCA1/2, BRIP1, CDK4/12, CHEK1/2, DSS1, EMSY, ERCC3, FANCA/D2, HDAC2, IDH1, LIG3/4, MDC1, MLH1/3, MRE11, NBN, PALB2, PRKDC, RAD50/51/54, XRCC6) who have progressed on previous ICI therapy (NCT04187833) [100]. Similarly, another phase II trial evaluates the efficacy of the combination of olaparib and pembrolizumab in treating patients with MM carrying the homologous recombination pathway gene mutation or alteration and who have progressed on previous immunotherapy, BRAF-targeting therapy, or both (NCT04633902). 

#### 3.6.9. ATR Inhibitors

Ataxia telangiectasia and Rad3-related protein kinase (ATR) is a crucial element of the cellular DNA damage response (DDR) in human cells [101]. ATR is a serine/threonine-specific protein kinase activated by replication stress resulting in single-stranded DNA breaks. Several studies have demonstrated that ATR inhibition is selectively toxic to tumor cells, with high levels of oncogene-induced replication stress [102,103,104,105]. Therefore, these studies provide the basis for the development of ATR inhibitors as antitumoral agents. Ceralasertib is a potent, selective, orally-administered inhibitor of ATR, with a good level of selectivity against PI3Ks and other phosphatidylinositol 3-kinase–related kinase family members, including mTOR, ATM, and DNA-PK [106,107]. In an open-label, phase I study, which aimed to assess the safety, pharmacodynamics, and preliminary efficacy of ceralasertib in combination with weekly paclitaxel in patients with refractory advanced solid tumors, 33 patients with melanoma, resistant to previous anti-PD1 therapy, were enrolled (NCT02630199). In the melanoma cohort, the ORR was 33.3%, the mPFS was 3.6 months, the median DOR was 9.9 months, and the mOS was 7.4 months [108]. Similarly, a phase II study (NCT03780608) was designed to assess the efficacy and safety of ceralasertib in combination with durvalumab in patients with MM who had progressed on anti-PD-1 therapy. Thirty MM patients exposed to previous anti-PD-1 therapy were enrolled (23 primary resistant). The ORR was 30.0% (9 PRs, 10 SDs, 10 PDs), DCR 63.3%, median PFS 7.1 months and mOS was 14.2 months [109]. 

Ongoing clinical studies with targeted therapy are listed in Table 5.

### 3.7. Combination of Intratumoral and Systemic Immunotherapy

#### 3.7.1. Oncolytic Virus

The administration of oncolytic viruses directly into the tumor microenvironment is a new possible approach to enhance tumor antigen recognition and increase T-cell response. Talimogene laherparepvec (T-VEC) is a genetically modified herpes simplex type 1 virus that expresses GM-CSF. It selectively infects and replicates in tumor cells leading to cell lysis and the releasing of GM-CSF. GM-CSF stimulates DCs, which successively process and present tumor antigens to cytotoxic T lymphocytes, producing a systemic tumor specific immune response. T-VEC has been approved in Europe for the treatment of unresectable IIIB, IIIC, and IV M1a stage melanoma with no bone, brain, lung, or other visceral disease, based on the results of the OPTiM phase 3 trial, which showed a durable response rate (lasting more than 6 months) and a significantly higher ORR following treatment with intralesional T-VEC in comparison with subcutaneous GM-CSF [110]. The final analysis also showed an improved mOS (23.3 months vs. 18.9 months in the T-VEC and GM-CSF arms, respectively, HR 0.79; *p* = 0.0494) [111]. Furthermore, T-VEC increases T-activation by improving antigen presentation and T-cell priming [112] and may increase the activity of anti-PD-1 therapy [110]. A phase II trial compared T-VEC plus ipilimumab with ipilimumab alone in patients with stages IIIB to IV melanoma. The combination demonstrated a durable lasting (≥6 months) ORR compared with ipilimumab alone (36.7 vs. 16.0%), responses were not limited to injected lesions. The CR rate was 21.4% vs. 6.0% in the ipilimumab alone group. CR was associated with a prolonged OS: 3-year OS rate was 100% for patients with CR and 52.3% for those with non-CR [113]. 

The MASTERKEY-265 phase Ib trial is a single-arm study that enrolled 21 patients who had unresectable or metastatic melanoma with injectable, measurable lesions and no prior systemic treatment to receive T-VEC plus pembrolizumab. At almost 5 years of follow-up, the median PFS and OS were not reached for patients treated with the combination. The CR rate remained at 43% (9/21 patients), and 92% of responders remained in response with improved OS observed in responders compared with non-responders [114]. In the corresponding randomized phase III trial, a total of 692 patients were randomized to receive T-VEC plus pembrolizumab or placebo plus pembrolizumab. Unfortunately, T-VEC plus pembrolizumab did not significantly improve the PFS or OS over the placebo plus pembrolizumab (NCT02263508) [115]. Two phase II trials are ongoing to assess the efficacy of T-VEC combined with pembrolizumab in patients with advanced melanoma (stage IIIB-IV) who experienced progression on prior PD1-therapy (NCT04068181¸ NCT02965716). 

Canerpaturev (C–REV) is another oncolytic virus, a spontaneous mutant of Herpes Simplex virus 1 (HSV-1). A phase II trial combined intratumoral injection of C-REV and ipilimumab in patients with pretreated advanced melanoma (NCT03153085) [116].

#### 3.7.2. Toll-like Receptor (TLR) 9 Agonist

TLR9 is a member of the Toll-Like-Receptor family, which is a class of proteins that play a key role in the immune system, inducing immune response to pathogen associated molecular patterns. TLR9 has demonstrated inducing potent antitumor responses, stimulating a potent innate and adaptive immune response that may augment the efficacy of ICI [49]. Tilsotolimod (IMO-2125) is a TLR9 agonist with robust immunostimulating activity: DCs activation, type I interferon response, CD8+ T-cell proliferation. ILLUMINATE-204 was a phase I/II study of the intratumoral administration of tilsotolimod with ipilimumab in patients with advanced melanoma who progressed on or after anti-PD-1 therapy [117]. The median OS was 21 months, and the ORR was 22.4%, including two CR and an additional 49% of patients had SD. The median duration of response was 11.4 months with seven out of 11 durable lasting (≥6 months) responses. Tumor reduction was seen in injected and non-injected lesions [118]. The phase III randomized, multicenter, open-label study ILLUMINATE 301 comparing the same combination with ipilimumab demonstrated a low ORR in patients with anti–PD-1 refractory advanced melanoma, missing the coprimary endpoint of this trial. The DCR observed with the combination was 34.5% versus 27.2% with ipilimumab monotherapy. In addition to the ORR, the study is exploring overall survival as a coprimary end point, but OS data have not been reported yet (NCT03445533). 

Despite these disappointing results, ILLUMINATE-206 is another ongoing open-label, multi-center, multicohort phase 2 study of approximately 30 patients with specific solid tumors, including melanoma, who are treated with the combination of tilsotolimod and nivolumab plus ipilimumab, exploring the efficacy of this combination (NCT03865082). 

CMP-001 is a new TLR-9 agonist consisting of an unmethylated CpG-A motif rich G10 oligodeoxynucleotide encapsulated in virus-like particles. In situ immunization with CMP-001 theoretically stimulate local tumor-associated plasmacytoid DCs, inducing type I interferon secretion and tumor antigen presentation to T cells and systemic antitumor T cell responses [119]. Promising preliminary results have been observed in patients previously treated with anti-PD-1 therapy patients with unresectable or MM using the combination of CMP-001 and pembrolizumab (NCT02680184), and in patients naive to PD-1 blockade with MM treatment with CMP-001 combined with nivolumab (NCT03618641). Phase II/III trials are ongoing to confirm the efficacy of this combination (NCT04698187, NCT04695977). 

SD-101 is another synthetic CpG TLR9 agonist. SYNERGY-001/KEYNOTE-184, a phase Ib/II trial, assessed the safety and preliminary efficacy of SD-101 combined with pembrolizumab in anti PD-1/L1 therapy-naive patients. SD-101 was tested as a 2 mg/lesion injection into one to four lesions and an 8 mg/lesion injection in one lesion. The ORR in 2 mg and 8 mg were 71% (CR 13%) and 49% (CR 7%), respectively, with responses in both injected and non-injected lesions, including visceral. The PFS was higher in the 2 mg group (NR vs. 10.4 months) than in the 8 mg group (HR 0.45, *p* = 0.036); and the 6-month OS was 98% vs. 92% [120]. 

PV-10 (10% rose bengal disodium for injection) is a small molecule autolytic immunotherapy. Intralesional injections can produce immunogenic cell death and induce tumor-specific reactivity in circulating T cells. Functional T-cell activity may be increased through combination with immune checkpoint blockade. PV-10-MM-1201 is a phase Ib/II trial of PV-10 combined with pembrolizumab for patients with checkpoint inhibitor naive or refractory advanced cutaneous melanoma. Among the naive and refractory population, the ORR was 67% and 31%, respectively. The median PFS was estimated at 11.7 months in the naive group [121]. An ongoing phase II study randomizes patients to receive PV-10 in combination with pembrolizumab or pembrolizumab alone (NCT02557321).

Ongoing studies are listed in Table 6.

## 4. Conclusions

Up to this point, checkpoint inhibition and targeted therapies have provided durable survival for a substantial number of patients with MM. However, many patients continue to experience disease progression, highlighting the need for new strategies to further improve outcomes. One strategy is to focus on applying PD-1 antibodies as a standard of care and adding other immune-modulating or microenvironment modulating agents such as anti-LAG3 antibodies or IL-2 agonist. Both combinations, which have recently demonstrated encouraging efficacy results and manageable safety profiles in previously untreated MM, are potential candidate to become new first line options for MM. In contrast, adoptive cell therapy has demonstrated to be effective in heavily pretreated MM, and especially in patients with primary resistance to prior anti-PD1 therapy. The strategy of combining BRAF-MEK inhibition and checkpoint inhibitors showed controversial results. Numerous clinical trials are investigating other novel therapeutic approaches, usually in combination with a standard of care ICI or BRAF-MEK inhibition. Identifying predictive biomarkers remains crucial for a personalized approach of treatment.

## Figures and Tables

**Table 1:** Novel immunecheckpoint inhbitors.

Agent	Class	Phase	Trial ID	Status on ClinicalTrials.gov (Accessed on 10 October 2021)
relatlimab + nivolumab (RELATIVITY-047)	anti-LAG3 + anti-PD-1	II/III	NCT03470922	active, not recruiting
relatlimab + nivolumab	anti-LAG3 + anti-PD-1	II	NCT03743766	recruiting
vibostolimab + pembrolizumab	anti-TIGIT + anti-PD-1	I/II	NCT04305054	recruiting
vibostolimab + pembrolizumab + quavonlimab	anti-TIGIT + anti-PD-1 + anti-CTLA-4	I/II	NCT04305041	recruiting

LAG3: Lymphocyte Activating Gene 3; PD-1: Programmed Death 1; TIM-3: T-cell immunoglobulin and mucin domain 3, TIGIT: T cell immunoreceptor with Ig and ITIM domains, CTLA-4: Cytotoxic T-Lymphocyte Antigen 4.

**Table 2:** Adoptive cell therapy.

Agent	Class	Phase	Trial ID	Status on ClinicalTrials.gov (Accessed on 10 October 2021)
Lifileucel	TILs	II	NCT02360579	active, not recruiting
TILs	TILs	III	NCT02278887	recruiting
ITIL-168	TILs	II	NCT05050006	recruiting
TILs + nivolumab	TILs + anti-PD-1	I	NCT04165967	recruiting
TILs + nivolumab	TILs + anti-PD-1	I	NCT03475134	recruiting
TILs + nivolumab	TILs + anti-PD-1	I/II	NCT03374839	recruiting
TILs + nivolumab	TILs + anti-PD-1	I/II	NCT03638375	recruiting

TILs: Tumor-infiltrating lymphocytes; PD-1: Programmed Death 1.

**Table 3:** Vaccines.

Agent	Class	Phase	Trial ID	Status on ClinicalTrials.gov (Accessed on 10 October 2021)
UV1 vaccine + ipilimumab	peptide vaccine + anti-CTLA-4	I/II	NCT02275416	active, not recruiting
UV1 vaccine + pembrolizumab	peptide vaccine + anti-PD1	I	NCT03538314	active, non recruiting
UV1 vaccine + nivolumab + ipilimumab	peptide vaccine + anti-PD1 + anti-CTLA-4	II	NCT04382664	recruiting
6MHP + NeoAg-mBRAF	peptide vaccine + neoantigen vaccine	I/II	NCT04364230	recruiting
neoantigen vaccine toripalimab + imiquimod 5% topical cream	neoantigen vaccine + anti-PD1 + topical cream	I	NCT04072900	recruiting
NeoVax + montanide + ipilimumab + nivolumab	neoantigen vaccine + anti-PD1 + anti-CTLA-4	I	NCT03929029	recruiting
TLPLDC	dendritic cells vaccine	I/II	NCT02678741	completed
mature dendritic cell vaccine + pembrolizumab	dendritic cells vaccine + anti-PD-1	I	NCT03092453	recruiting
BNT111 + cemiplimab	mRNA vaccine + anti-PD1	II	NCT04526899	recruiting

CTLA-4: Cytotoxic T-Lymphocyte Antigen 4; PD-1: Programmed Death 1, BRAFi: BRAF inhibitor; MEKi: MEK inhibitor.

**Table 4:** Engineered TCR therapy and Interleukin-2 studies.

Agent	Class	Phase	Trial ID	Status on ClinicalTrials.gov (Accessed on 10 October 2021)
MB-CART20.1	CAR-T cells	I (15 pts)	NCT03893019	recruiting
IL13Ralpha2 CAR T cells	CAR-T cells	I (24 pts)	NCT04119024	recruiting
GPA-TriMAR-T	CAR-T cells	I (6 pts)	NCT03649529	recruiting
IMA202 product	TCR engineered T cells	I (15 pts)	NCT03441100	recruiting
IMA203 product +/− atezolizumab	TCR engineered T cells +/− anti PD-1	I (42 pts)	NCT03686124	recruiting
Bempegaldesleukin + nivolumab	IL-2 + anti-PD-1	III	NCT03635983	recruiting
Nemvaleukin Alfa	IL-2	II	NCT04830124	recruiting
Hu14.18-IL2 + RT + nivolumab + ipilimumab	IL-2 + anti-PD-1 + anti-CTLA-4	I/II	NCT03958383	recruiting
IL2 + ipilimumab followed by nivolumab	IL-2 + anti-CTLA-4 + anti-PD-1	II	NCT04562129	recruiting

CAR T-cells: Chimeric Antigen Receptor T cells; IL-2: Interleukin 2; PD-1: Programmed Death 1; RT: radiotherapy; CTLA-4: Cytotoxic T-Lymphocyte Antigen 4.

**Table 5:** Targeted Therapy.

Agent	Class	Phase	Trial ID	Status on ClinicalTrials.gov (Accessed on 10 October 2021)
FCN-159	MEK inhibitor	I	NCT03932253	recruiting
HL-085	MEK inhibitor	I/II	NCT03973151	recruiting
belvarafenib + cobimetinib + atezolizumab	RAF inhibitor + MEKi + anti-PD-L1	I	NCT0483805	recruiting
LXH254 + LTT462 or trametinib or ribociclib	BRAF and CRAF inhibitor + ERK inhibitor or MEKi or CDK4/6 inhibitor		NCT0483805	recruiting
palbociclib + encorafenib + binimetinib	CDK4/6 inhibitor + BRAFi + MEKi	Ib/II	NCT04720768	recruiting
ulixertinib	ERK inhibitor	expanded access	NCT04566393	available
domatinostat + pembrolizumab	HDAC inhibitor + anti-PD-1	Ib/II	NCT03278665	recruiting
entinostat + pembrolizumab	HDAC inhibitor + anti-PD-1	II	NCT03765229	recruiting
HBI-8000 + nivolumab	HDAC inhibitor + anti-PD-1	III	NCT04674683	recruiting
lenvatinib + pembrolizumab	anti-VEGFR + anti-PD-1	III	NCT03820986	active, not recruiting
axitinib + nivolumab	anti-VEGFR + anti-PD-1	II	NCT04493203	recruiting
bevacizumab + ipilimumab	anti-VEGF + anti-CTLA-4	II	NCT01950390	active, non recruiting
bevacizumab + atezolizumab	anti-VEGF + anti-PD-L1	II	NCT04356729	recruiting
pembrolizumab or dabrafenib/trametinib + bemcentinib	anti-PD-1 or BRAFi/MEKi + Axl inhibitor	Ib/II	NCT02872259	recruiting
niraparib	PARP inhibitor	II	NCT03925350	recruiting
olaparib + pembrolizumab	PARP inhibitor + anti-PD-1	II	NCT04633902	recruiting
talazoparib + nivolumab	PARP inhibitor	II	NCT04187833	recruiting

PD-1: Programmed Death 1; BRAFi: BRAF inhibitor; MEKi: MEK inhibitor; VEGF: vascular endothelial growth factor, VEGFR: vascular endothelial growth factor receptor; PARP: Poly-ADP-ribose polymerase; PD-L1: Programmed Death Ligand-1.

**Table 6:** Intra-tumoral and systemic therapy.

Agent	Class	Phase	Trial ID	Status on ClinicalTrials.gov (Accessed on 10 Ocober 2021)
Talimogene laherparepvec + pembrolizumab	modified oncolytic HSV 1 + anti-PD-1	II	NCT02965716	active, non recruiting
Talimogene laherparepvec + pembrolizumab	modified oncolytic HSV 1 + anti-PD-1	II	NCT04068181	active, non recruiting
CMP-001 + nivolumab	TLR9 agonist + anti-PD-1	II	NCT04698187	recruiting
CMP-001 + nivolumab	TLR9 agonist + anti-PD-1	II/III	NCT04695977	recruiting
PV-10 + pembrolizumab	small molecule autolytic immunotherapy + anti-PD-1	I/II	NCT02557321	recruiting

PD-1: Programmed death 1; HSV1: Herpes Simplex Virus 1; TLR9: Toll Like Receptor 9.
